# Draft genome sequence of the HP60 serovar of *Avibacterium paragallinarum*

**DOI:** 10.1128/mra.00146-25

**Published:** 2025-09-29

**Authors:** Azil Coertzen, Mariana Erasmus, Samantha J. McCarlie, Robert R. Bragg

**Affiliations:** 1 The Centre for Mineral Biogeochemistry (CMBG), University of the Free State 37702, Bloemfontein, South Africa; Loyola University Chicago, Chicago, Illinois, USA

**Keywords:** *Avibacterium paragallinarum*, reference serovars, comparative analysis

## Abstract

Infectious coryza, caused by *Avibacterium paragallinarum*, poses a challenge for poultry farms due to the inefficacy of vaccines. The genome of *A. paragallinarum* serovar HP60, which belongs to the C-serogroup, was sequenced to enable comparative analysis with other serovar genomes to identify the similarities and differences between the genomes.

## ANNOUNCEMENT

Poultry remains an affordable protein source; however, infectious coryza caused by *Avibacterium paragallinarum* (1–3) can pose a serious threat, leading to a decline in egg production (4). This bacterium is divided into three (A, B, and C) serogroups, with nine reference serovars (5–7). Identifying the serovars is crucial, as vaccines provide no cross-protection, particularly within serogroup C (4, 8). Therefore, the genome of reference HP60 serovar (C-4) will be utilized in a comparative analysis with the other reference serovar genomes to detect similar and different genes between the genomes to understand the serovars better.

The HP60 serovar, originally isolated from an infected chicken in Australia and identified as a C-4 reference serovar, was stored at the University of the Free State. It was cultured on blood agar plates with the colony feeder *Staphylococcus epidermidis* to supply nicotinamide adenine dinucleotide (NAD^+^). This method was completed under microaerophilic conditions at 37°C for 24 hours. Tryptic soy broth was supplemented with 5% NAD^+^ and inoculated with the serovar. The deoxyribonucleic acid (DNA) was extracted using the Labuschagne and Albertyn protocol (9). The DNA quantity and quality were checked with the Fluorometer Qubit 4 (Invitrogen).

Species-specific polymerase chain reaction (PCR) (10) and 16S ribonucleic acid (RNA) PCR confirmed that no contamination of the colony feeder occurred and that the genomic DNA belongs to *A. paragallinarum*. The genomic DNA was prepared following the manual with the Nextera XT preparation kit, then sent for next-generation sequencing on the Illumina MiSeq platform at the Inqaba Biotechnical Industries (Pretoria, South Africa). Paired-end reads (2 × 150 bp) were produced, and 2,203,752 reads were generated, where the quality was assessed using the FastaQC version 0.12.0 (11) and PRINSEQ version 0.20.4 (12), both following the default parameters. These reads were then submitted to the SPAdes version 4.2.0 genome assembler (13) using the assembly graph construction approach with the default parameters to produce the data given in Table 1.

**TABLE 1:** Summary statistics for the HP60 (C-4) assembly

Serovar	Base pairs	Number of contigs	Genome coverage	G + C content (%)	*N*_50_ value	*L*_50_ value
HP60	2,427,563 bp	305	30×	40.9	26,856	32

The 16S gene from the HP60 contigs was annotated using the Rapid Annotation using Subsystems Technology server (14). The rRNA/ITS database was used in the Basic Local Alignment Search Tool from the National Center for Biotechnology Information database (15). This search confirmed that this genome sequence matched the 16S rRNA gene of the reference HP60 serovar (KC951276.1), with a query cover of 92% and percent identity of 99.92%.

## Data Availability

The genome assembly of HP60 has been uploaded to the NCBI database as a BioProject PRJNA1080358, GenBank accession number (GCA_037080095.1), and a BioSample SAMN40127135. It is also available in the GenBank database with the project accession number JBANLW000000000 and the accession number JBANLW01. This available sequence is not annotated. The MiSeq data have been included in the Sequence Read Archive (SRA) under accession number SRR32381724. The parameters of the genome assembly have been placed in Table 1.
